# Surface Charge Transfer Doping of MoS_2_ Monolayer by Molecules with Aggregation-Induced Emission Effect

**DOI:** 10.3390/nano12010164

**Published:** 2022-01-04

**Authors:** Ruihao Sun, Shiyu Sun, Xiu Liang, Hongyu Gong, Xingshuang Zhang, Yong Li, Meng Gao, Dongwei Li, Guanchen Xu

**Affiliations:** Key Laboratory for High Strength Lightweight Metallic Materials of Shandong Province (HM), Advanced Materials Institute, Qilu University of Technology (Shandong Academy of Sciences), Jinan 250014, China; srh1645@163.com (R.S.); sysun0313@163.com (S.S.); xliang@sdas.org (X.L.); hygong@sdas.org (H.G.); xszhang@qlu.edu.cn (X.Z.); yongli@sdas.org (Y.L.); mgao@sdas.org (M.G.); dwli@sdas.org (D.L.)

**Keywords:** MoS_2_ monolayer, surface charge transfer doping, aggregation-induced emission, photoluminescence, density functional theory

## Abstract

Surface charge transfer doping has attracted much attention in modulating the optical and electrical behavior of 2D transition metal dichalcogenides (TMDCs), where finding controllable and efficient dopants is crucial. Here, 1,1,2,2-tetraphenylethylene (TPE) derivative molecules with aggregation-induced emission (AIE) effect were selected as adjustable dopants. By designing nitro and methoxyl functional groups and surface coating, controlled p/n-type doping can be achieved on a chemical vapor deposition (CVD) grown monolayer, MoS_2_. We investigated the electron transfer behavior between these two dopants and MoS_2_ with fluorescence, Raman, X-ray photoelectron spectra and transient absorption spectra. 1,1,2,2-Tetrakis(4-nitrophenyl)ethane (TPE-4NO_2_) with a negative charge aggregation can be a donor to transfer electrons to MoS_2_, while 1,1,2,2-Tetrakis(4-methoxyphenyl)ethane (TPE-4OCH_3_) is the opposite and electron-accepting. Density functional theory calculations further explain and confirm these experimental results. This work shows a new way to select suitable dopants for TMDCs, which is beneficial for a wide range of applications in optoelectronic devices.

## 1. Introduction

Transition metal dichalcogenides (TMDCs) have attracted much attention as promising layer-like materials. When transformed from 3D bulk to a 2D monolayer, they possess a direct bandgap and exceptional optoelectronic properties [1,2,3,4,5]. Among them, 2D TMDCs with semiconductor properties represented by the monolayer MoS_2_ and WS_2_ show excellent electrical and optical properties, high switching ratio and large carrier mobility, which make them valuable for a wide range of applications in logic circuits, photodetectors, photovoltaic circuits, light-emitting diodes and other optoelectronic devices [6,7,8]. Semiconductor doping works as an effective way to prepare homogeneous/heterogeneous junctions for logic circuits and has recently become increasingly popular. At present, the doping engineering used in 2D TMDCs includes substitution doping [9,10], intercalation doping [11], electrostatic doping [12] and surface charge transfer doping (SCTD). Among these techniques, SCTD has the advantages of being effective with no lattice damage. Because of the difference in work function or electronegativity between the surface dopant and the 2D material, charge (electron or hole) transfer in one direction is generated between the two interfaces [13,14]. Then, the charge density and carrier mobility of the device can be effectively adjusted. In the past, the use of H_2_O, O_2_ [15], NO_2_, NH_3_ [16] and K [17] was explored for doping TMDCs by SCTD. The complex and unstable doping process may limit their prospects. Organic dopants offer rich and powerful functional groups, which can remarkably tune the optical and electrical properties of TMDCs. F_4_TCNQ [18] and BV [19] have been shown to allow the p-type/n-type doping of monolayers of MoS_2_. However, the molecules are single and uncontrollable. Thus, finding controllable and efficient dopants is crucial. Molecules with aggregation-induced emission (AIE) effect have the advantages of large, conjugated structures, high symmetry and strong scalability, which can allow them to serve as an electron donor or acceptor [20,21]. In this paper, we chose the TPE molecule [22] as a backbone to realize controllable and efficient p/n doping of the monolayer MoS_2_ based on molecular structure design. We present the surface charge transfer doping of the chemical vapor deposition (CVD)-grown monolayer MoS_2_ by using designed AIE molecules formulated in low concentration solutions as organic dopants to selectively tune the optical and electrical properties of the CVD-grown monolayer MoS_2_. The X-ray photoelectron spectroscopy (XPS), photoluminescence (PL) spectroscopy and transient absorption (TA) spectroscopy results showed that the surface charge transfer mode between AIE molecules and monolayer MoS_2_ could be adjustable by just changing the electronegativity of functional groups in TPE. The evolution of the band structure and density of states (DOS) of TPE-based molecule doped MoS_2_ monolayers were also confirmed by density functional theory (DFT). This work finds AIE dopants as a new way to dope 2D TMDCs materials.

## 2. Materials and Methods

### 2.1. CVD Growth of MoS_2_

The MoOx growth precursors were prepared by electrochemical anodization of Mo foils. The anodization was performed at room temperature at 0.5 V for 30 min. After that, the oxidized Mo foils were rinsed with water and then dried naturally. The SEM image of the oxidized Mo foil is shown in Figure S1. In order to synthesize MoS_2_ flakes, the Mo foil was arched and placed on a 300 nm SiO_2_/Si substrate, which was then loaded into the center of the furnace. Sulfur powder (Alfa-Aesar, Ward Hill, MA, USA, 99.999%, 1.0 g) was placed at the upstream entrance of the furnace. Next, 200 sccm of Ar was introduced and for 20 min to remove the oxygen and moisture from the system. The furnace was then heated to 850 °C for 43 min under an Ar flow at 100 sccm. Sulfur powders were heated by an individual heating belt at ≈150 °C when the temperature of the furnace reached 630 °C. Then, the furnace temperature was kept constant at 850 °C for 15 min, then cooled naturally to room temperature. Heating the sulfur powder with the heating belt was stopped when the furnace cooled to 400 °C [23].

### 2.2. Synthesis of AIE Molecules

1,1,2,2-Tetrakis(4-nitrophenyl)ethane (TPE-4NO_2_) was synthesized according to the literature procedure [24]. The ^1^H NMR spectrum was consistent with that previously reported. According to the synthesis steps, TPE (1,1,2,2-tetraphenylethylene) was first synthesized. Zinc powder (30 g, 459 mmol) and THF (anhydrous, 150 mL) were added to a 500 mL three-neck flask. The reaction was degassed, flushed with dry nitrogen and cooled to −40 °C in a dry ice/CH_3_CN bath before titanium(IV) chloride (25 mL, 228 mmol) was added dropwise. The reaction mixture was warmed to room temperature first and then heated to 90 °C for 2 h with stirring. The reaction was cooled to 0 °C before benzophenone (23 g, 126 mmol) was added. It was then heated to 90 °C again with stirring overnight, cooled and quenched using potassium carbonate (10% wt) aqueous solution (400 mL). The precipitate was collected by filtration and air-dried overnight. The solid was dissolved in CH_2_Cl_2_ (200 mL), and insoluble residues were removed by filtration. The solvent of the filtrate was removed under reduced pressure to afford a white solid (19.85 g, 60 mmol) as the desired compound of 95% yield. ^1^H NMR (500 MHz, CDCl_3_, 298 K): δ = 7.03–7.06 (m, 8H), 7.09–7.13 (m, 12H). Then, TPE-4NO_2_ was synthesized in an ice bath, and acetic acid (40 mL, 700 mmol) and fuming nitric acid (40 mL, 968 mmol) were added to a 250 mL round-bottom flask. TPE (5.0 g, 15 mmol) was added in small portions over a 20 min period before the reaction was warmed to room temperature with stirring for 3 h. The reaction mixture was poured into ice water (300 mL) and the yellow precipitate was collected by filtration, washed with an excess of water and air-dried, affording a light-yellow powder (6.6 g, 11 mmol) of 85% yield. ^1^H NMR (500 MHz, CDCl_3_, 298 K): δ = 8.08 (m, 8H), 7.19 (m, 8H).

1,1,2,2-Tetrakis(4-methoxyphenyl)ethane (TPE-4OCH_3_) was synthesized in chiral monomethoxybenzophenone (5.82 g, 20 mmol), and zinc dust (5.69 g, 60 mmol) was vigorously stirred in THF (200 mL) under Ar. Titanium tetrachloride (3.3 mL, 30 mmol) was added dropwise at 0 °C, and the resulting mixture was refluxed for 10 h. The crude mixture obtained was poured in aqueous sodium carbonate and ether (2 × 200 mL) was added to extract the organic material. The obtained organic phase was washed with brine and water and dried using sodium sulfate. The residue obtained after evaporation of the filtrate was purified by silica gel column chromatography, eluting with hexane-ethyl acetate (25:1). The obtained material (4.12 g, 77%) was further purified by using recrystallization in ethanol to give 3.51 g (66%, E/Z > 98) of the gas chromatographically pure (E)-isomer. ^1^H NMR (500 MHz, CDCl_3_, 298 K): δ = 6.92 (m, 8H), 6.64 (m, 8H), 3.75 (m, 12H) [25].

### 2.3. Characterizations

SEM images were taken with JEOL JSM-7610FPlus at 15 kV. Optical images were captured with an Olympus BX 53 M microscope. TEM images and SAED patterns were acquired with an FEI JEM 2100F at 200 kV. AFM images were taken on the Bruker Bioscope Resolve in ScanAsyst. Raman and PL measurements were carried out with a Horiba SMART Raman system at 532 nm laser excitation with a power of 50 mW. The Si peak at 520.7 cm^−1^ was used for calibration in the data analysis of Raman and PL spectra. The fluorescence spectra were obtained using a FS5 fluorescence spectrofluorometer under 365 nm excitation. XPS was measured with Thermo ESCALAB 250XI with Al Kα. TAS was performed with a diode-pump Yb medium femtosecond laser with 190 fs pulses at 1030 nm and a repetition rate of 100 kHz. The laser was converted to varied wavelengths from 580 nm to 720 nm through an optical parametric amplifier (OPA). ^1^H-NMR spectra were acquired on a Bruker 400 MHz NMR spectrometer at room temperature using CDCl_3_ as the solvent.

### 2.4. DFT Calculation

All calculations were performed within the framework of density function theory, using the method of projector-enhanced plane wave method in the Vienna ab initio simulation package [26]. The exchange-correlation potential was obtained using the generalized gradient approximation proposed by Perdew, Burke and Ernzerhof [27]. The cutoff energy of the plane wave was set to 400 eV. In the iterative solution of the Kohn-Sham equation, the energy criterion was set to 10^−5^ eV. A 2 × 2 × 1 k-mesh was used for Brillouin zone integration. All the structures were relaxed until the residual forces on the atoms decreased to less than 0.05 eV/Å.

## 3. Results and Discussion

**Figure 1:**
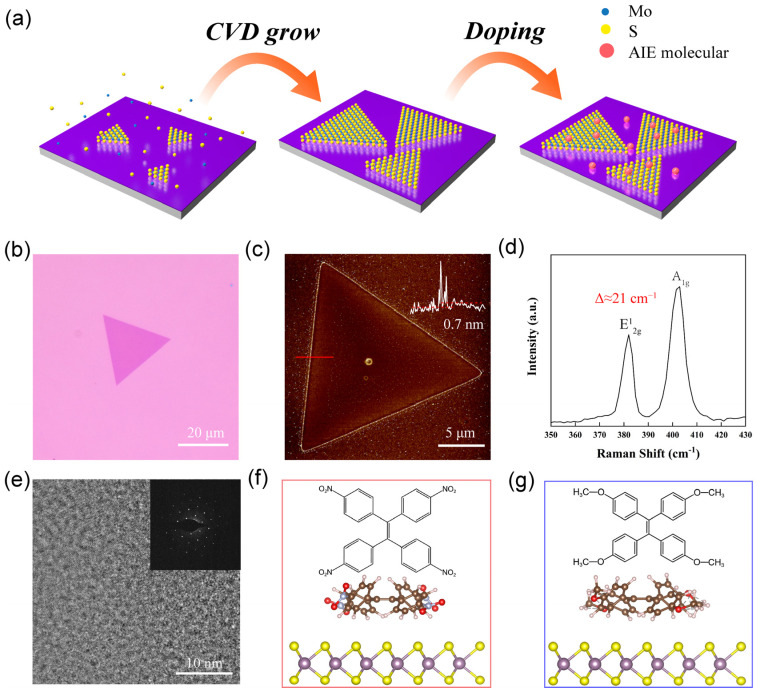
(**a**) Schematic diagram of synthesized monolayer MoS_2_ and doping. The monolayer of MoS_2_ is grown by the CVD method, and then the grown MoS_2_ is immersed in the AIE molecules solution for doping. (**b**) Optical microscope image of MoS_2_. (**c**) AFM image of MoS_2_. (**d**) Raman spectra of MoS_2_. (**e**) TEM image of MoS_2_. The inset is SAED patterns. (**f**) Structural formula of the TPE-4NO_2_ molecule and the atomic structure of a single TPE-4NO_2_ molecule attached to a single layer of MoS_2_. (**g**) Structural formula of the TPE-4OCH_3_ molecule and the atomic structure of a single TPE-4OCH_3_ molecule attached to a single layer of MoS_2_.

The schematic diagram of the synthesis process and doping strategy is shown in Figure 1a. The monolayer MoS_2_ was first grown on a SiO_2_/Si substrate by chemical vapor deposition (CVD) [23]. The obtained MoS_2_ shows perfectly a triangular shape with a length size at ~20 μm and a monolayer thickness of ~0.7 nm on an SiO_2_/Si substrate, which was evidenced by optical microscopy image and atomic force microscopy (Figure 1b,c). Raman spectra of as-synthesized MoS_2_ are shown in Figure 1d. The spacing between two characteristic peaks (A_1g_ mode ~403 cm^−1^ and E^1^_2g_ mode ~382 cm^−1^) was approximately 21 cm^−1^, which further confirms that the as-obtained MoS_2_ was a monolayer with a 2H phase [28,29]. Their high crystallinity was confirmed by transmission electron microscopy and selected area electron diffraction. Here, two TPE derivatives with AIE effect were synthesized. One was TPE-4NO_2_ obtained by introducing the strong electron-withdrawing group –NO_2_ on the basis of TPE, and the other was TPE-4OCH_3_ obtained by introducing the strong electron-donating group –OCH_3_ (Figure 1f,g). The ^1^H-NMR spectra in Figure S2 show that the high-quality and pure monolayer MoS_2_ and TPE derivative molecules (TPE-4NO_2_ and TPE-4OCH_3_) were well-prepared for subsequent works. The monolayer MoS_2_ was modified by immediately immersing it in dichloromethane solutions of AIE molecules with the concentration setting at 1 μmol/mL for 1 h. After doping, the surface of MoS_2_ became rough (Figure S3), demonstrating that the AIE molecules were successfully adsorbed on the surface of MoS_2_.

**Figure 2:**
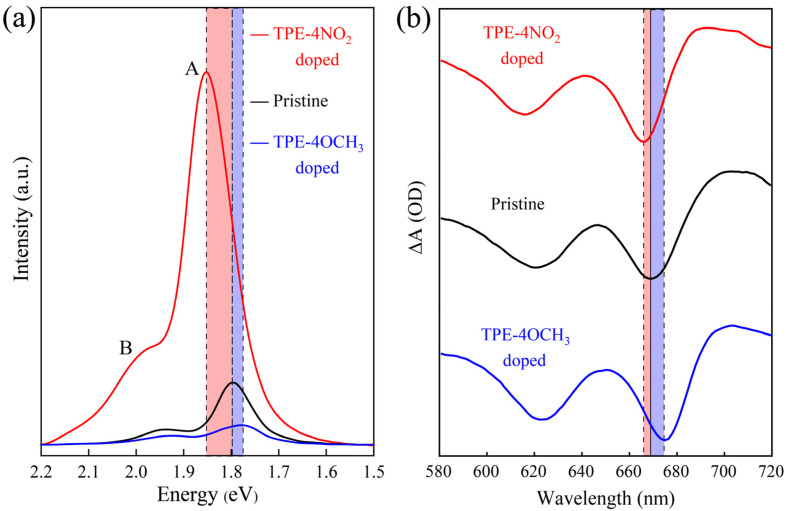
PL spectra (**a**) and transient absorption spectra (**b**) of pristine and doped MoS_2_ samples.

The optical properties of the TPE-4NO_2_ and TPE-4OCH_3_ molecules-doped monolayer MoS_2_ were investigated by photoluminescence (PL) spectra and transient absorption spectra. As shown in Figure 2a, two broad bands of A and B excitons appear in the PL spectra of the as-obtained samples, which correspond to the spin-orbit coupling of the valence band (direct excitonic transition). After doping the monolayer MoS_2_ with TPE-4NO_2_, the peak intensity was greatly enhanced, while the position of the peaks showed a significant blue shift. Moreover, after doping the monolayer MoS_2_ with TPE-4OCH_3_ molecules, the peak intensity was substantially quenched, while the position of the PL peaks underwent a substantial red shift. The effect of dichloromethane solvent on the doping process was negligible (Figure S4). When the doped sample was immersed in dichloromethane solvent, the dopant molecules were desorbed from the surface of MoS_2_ and dissolved into dichloromethane, at which time the PL properties of MoS_2_ were close to the original behavior (Figure S5), which is an advantage of surface charge transfer doping. This property can be used to adjust the optoelectronic properties of MoS_2_ more precisely. The PL of pure TPE-derived molecules in Figure S6 has a negligible contribution to the overall PL intensity, indicating that PL enhancement or quenching results from electron transfer rather than from the fluorescence contribution of TPE-derived molecules. Figure 2b shows transient absorption spectra (TAS) obtained by the pump-probe measurement at the 400 nm pump wavelength with a delay of 0.4 ps. Before and after doping, two strong bleach signals are detected. Two distinct bands of ground-state bleaching signals appear at the positions of the A and B exciton resonance absorption peaks, which are due to the reduction of ground-state absorption caused by conduction band filling during exciton formation [30]. The obvious blue shift of two bleaching peaks is caused by the strong multibody effect between high concentration carriers. This effect makes the center of gravity of the carrier population increase in the direction of energy increase in the conduction band and valence band, respectively, resulting in the blue shift of the peak position [31,32]. Correspondingly, the red shift of the bleaching peak of the TPE-4OCH_3_-doped monolayer MoS_2_ was consistent with the peak position change in the PL spectra.

**Figure 3:**
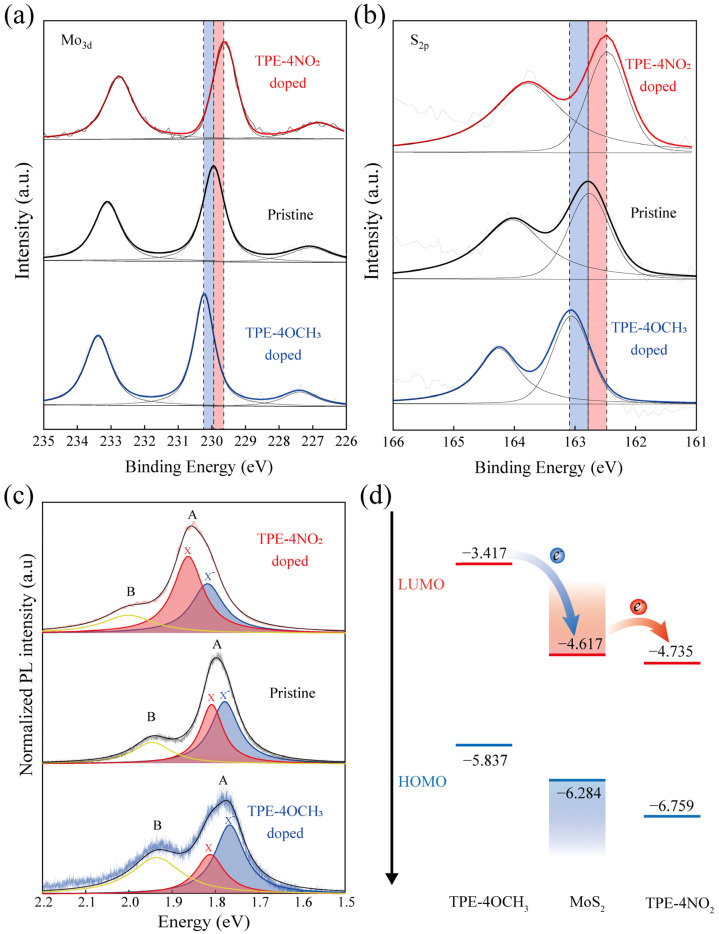
XPS spectra of Mo_3d_ (**a**) and S_2p_ (**b**) for pristine and doped MoS_2_ samples. (**c**) Normalized PL intensity for pristine and doped samples of monolayer MoS_2_ by the fitting of Lorentz peaks. (**d**) Energy band structure of MoS_2_, TPE-4OCH_3_ and TPE-4NO_2_.

The X-ray photoelectron spectra (XPS) show the charge transfer phenomenon during the doping process. Figure 3a,b shows the XPS spectra of the Mo_3d_ and S_2p_ orbitals in pristine and doped MoS_2_ samples. The spectral shapes of Mo_3d_ and S_2p_ elements are almost identical between the samples, all peak positions shifted from approximately 0.2~0.3 eV, and the Raman spectra after doping did not change substantially (Figure S7). These results suggest that physical adsorption occurs between the doped molecules and the monolayer MoS_2_. The OM images and AFM images of the as-modified MoS_2_ also show the same morphology in Figure S3. However, after the TPE-4NO_2_ doping treatment, the peak positions were shifted to lower binding energy, indicating a shift of the valence band toward the Fermi level, as expected to produce p-type doping. On the other hand, after the TPE-4OCH_3_ doping treatment, the peak positions shifted to higher binding energy, demonstrating that the conduction band moves downward to the Fermi level, producing an n-type doping feature [33,34,35].

In order to better understand the modulated mechanism of optical properties for the doped monolayer MoS_2_, the PL spectra were fitted into three Lorentz peaks in Figure 3c: the trion peak (X^−^), exciton peak (X) and B exciton peak. The emission intensities of X^−^ and X for pure MoS_2_ are basically the same. After TPE-4NO_2_ doping, the proportion of X in TPE-4NO_2_/MoS_2_ is significantly increased, which shows that the export of electrons leads to the production of more X with higher energy and promotes a substantial increase in the intensity of peak A. In contrast, after TPE-4OCH_3_ doping, X^−^ in TPE-4OCH_3_/MoS_2_ occupies the dominant position, and many electrons migrate in, which reduces the proportion of X, which leads to quenching [18,36]. Hence, we propose the energy band structure with molecules and a monolayer of MoS_2_ in Figure 3d, where the electrons jump from the CB of MoS_2_ to the LOMO of TPE-4NO_2_, and TPE-4NO_2_ accepts the electrons from MoS_2_ to create holes in MoS_2_, which achieves p-type doping. Meanwhile, MoS_2_ accepts the LUMO from TPE-4OCH_3,_ which causes the electrons to aggregate inside it, giving rise to the n-type doping feature.

**Figure 4:**
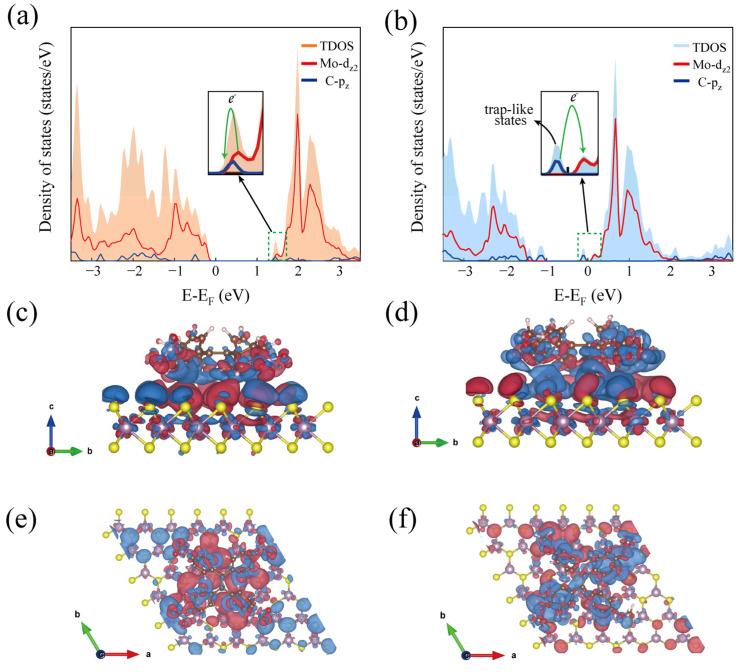
TDOS and PDOS of TPE-4NO_2_ doped (**a**) and TPE-4OCH_3_ doped (**b**). Front (**c**) and top views (**e**) of the charge density difference of MoS_2_/TPE-4NO_2_. Front (**d**) and top views (**f**) of the charge density difference of MoS_2_/TPE-4OCH_3_. Red and blue isosurfaces represent the charge accumulation (i.e., a gain of electron density) and depletion (i.e., a loss of electron density) in the system, respectively.

Moreover, density functional theory (DFT) calculations were conducted to further understand the modulatory mechanism. Figure 4a demonstrates the TDOS and PDOS after TPE-4NO_2_ doping, and it is clearly seen that under the CB, the Mo d_z2_ and C p_z_ orbitals are in close contact, thus, greatly promoting the electron transfer efficiency, which is the primary reason for the significant enhancement of PL. In Figure 4b, the doping of TPE-4OCH_3_ leads to the appearance of new DOS near the VB and CB due to the C pz orbital of the TPE-4OCH_3_ molecule. Furthermore, “trap-like” states appear, which prevent the complexation of electrons and holes and, thus, lead to PL quenching [37]. This result is consistent with the electronic band structure in Figure S8. In addition, the VB after TPE-4NO_2_ doping is closer to the Fermi energy level, while the CB after TPE-4OCH_3_ doping is closer to the Fermi energy level, and the results of this calculation agree with the experimental XPS results. Figure 4c,e shows the differential charge density diagrams after TPE-4NO_2_ doping in the front view (vector a) and top view (vector c). This figure visually shows that electrons are transferred from MoS_2_ to TPE-4NO_2_, and a large number of electrons are gathered around the molecule (red equivalent surface), while a large number of holes are left on MoS_2_ (blue equivalent surface), indicating the transformation of MoS_2_ into a p-type semiconductor. Figure 4d,f shows the differential charge diagram of TPE-4OCH_3_ after doping, with electrons shifting from TPE-4OCH_3_ to MoS_2_ and MoS_2_ to an n-type semiconductor [38]. This result demonstrates the ability of these two molecules to dope the monolayer MoS_2_ stably and effectively and transform MoS_2_ into a p-type/n-type semiconductor.

## 4. Conclusions

In summary, we designed and synthesized AIE effective molecules of TPE-4NO_2_ and TPE-4OCH_3_ for the controlled doping of the CVD-grown monolayer MoS_2_. After doping with a p-type dopant, TPE-4NO_2_, the PL intensity was drastically increased with enhanced exciton X and the peak was blue shifted, which was remarkably opposite to TPE-4OCH_3_. Different effects of the two dopants were also observed in XPS and TAS. DFT calculations demonstrated that the originations of p- and n-doping were dedicated from the framework of TPE with the AIE effect and their functional groups. Dopant selection provides a new strategy for tuning TMDCs, which will be expected to pave the way for their use in optical and electronic devices.

## Data Availability

Data is available upon the request from the corresponding author.

## Supplementary Materials

The following supporting information can be downloaded at: https://www.mdpi.com/article/10.3390/nano12010164/s1, Figure S1: SEM images of (a) pure Mo foil, (b) the growth precursor of MoOx was prepared by electrochemical anodization of Mo foil. Figure S2: ^1^H NMR spectrum of (a) TPE, (b) TPE-4NO_2_ (c) TPE-4OCH_3_ in CDCl_3_. Figure S3: (a–d) Images of OM and AFM before and after TPE-4NO_2_ doping. (e–h) Images of OM and AFM before and after TPE-4OCH_3_ doping. Figure S4: PL spectra of monolayer MoS_2_ before and after soak in DCM for 12 h. Figure S5: PL spectra of TPE-4NO_2_ (a) and TPE-4OCH_3_ (b) doped of immersion in dichloromethane (DCM) 12 h. Figure S6: (a–c) PL spectra of TPE, TPE-4NO_2_ and TPE-4OCH_3_ molecules. (d–f) Fluorescence spectra TPE, TPE-4NO_2_ and TPE-4OCH_3_ molecules, excitation wavelength is at 365 nm. Figure S7: Raman spectra of TPE-4NO_2_ (a) and TPE-4OCH_3_ (b) doped. Figure S8: The electronic band structure of (a) the monolayer MoS_2_, (b) MoS_2_/TPE-4NO_2_, (c) MoS_2_/TPE-4OCH_3_.
