# Supplementary material for: Surface Charge Transfer Doping of MoS2 Monolayer by Molecules with Aggregation-Induced Emission Effect
Source: Nanomaterials (Basel). 2022 Jan 4;12(1):164. doi: 10.3390/nano12010164 (PMC8746604; doi:10.3390/nano12010164)
Supplement: Supplementary file 1 [file nanomaterials-12-00164-s001.zip › nanomaterials-1517678-supplementary.pdf]

Supporting Information

Surface Charge Transfer Doping of MoS_2 Monolayer by Molecules with Aggregation-Induced Emission Effect

Ruihao Sun, Shiyu Sun, Xiu Liang, Hongyu Gong, Xingshuang Zhang, Yong Li, Meng Gao, Dongwei Li and Guanchen Xu *

Key Laboratory for High Strength Lightweight Metallic Materials of Shandong Province (HM), Advanced Materials Institute, Qilu University of Technology (Shandong Academy of Sciences), Jinan 250014, China; srh1645@163.com (R.S.); sysun0313@163.com (S.S.); xliang@sdas.org (X.L.); hygong@sdas.org (H.G.); xszhang@qlu.edu.cn (X.Z.); yongli@sdas.org (Y.L.); mgao@sdas.org (M.G.); dwli@sdas.org (D.L.)

* Correspondence: gcxu@sdas.org

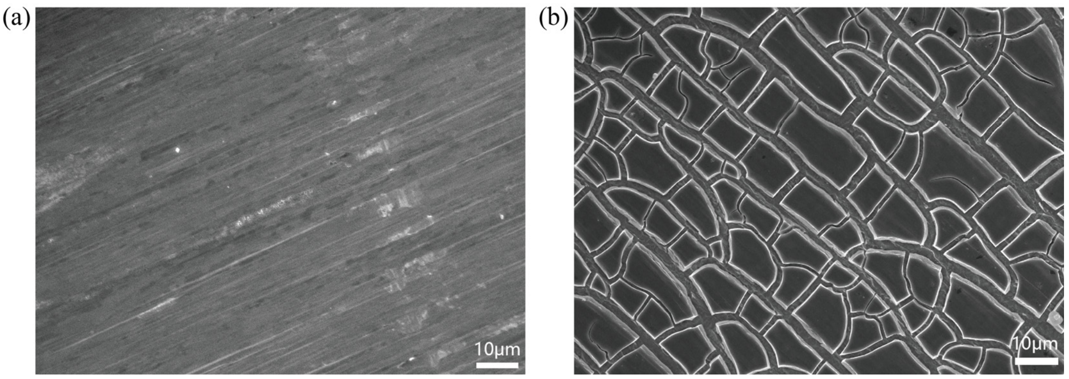

Figure S1 SEM images of (**a**) Pure Mo foil (**b**) The growth precursor of MoOx was prepared by electrochemical anodization of Mo foil.

(a)

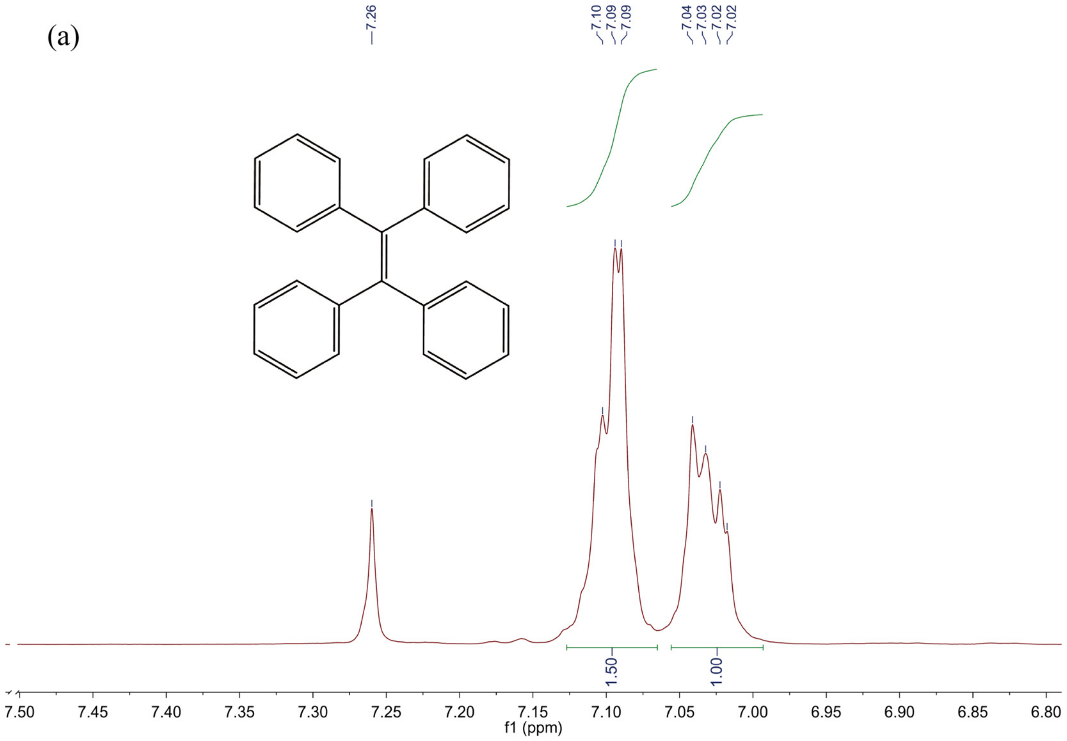

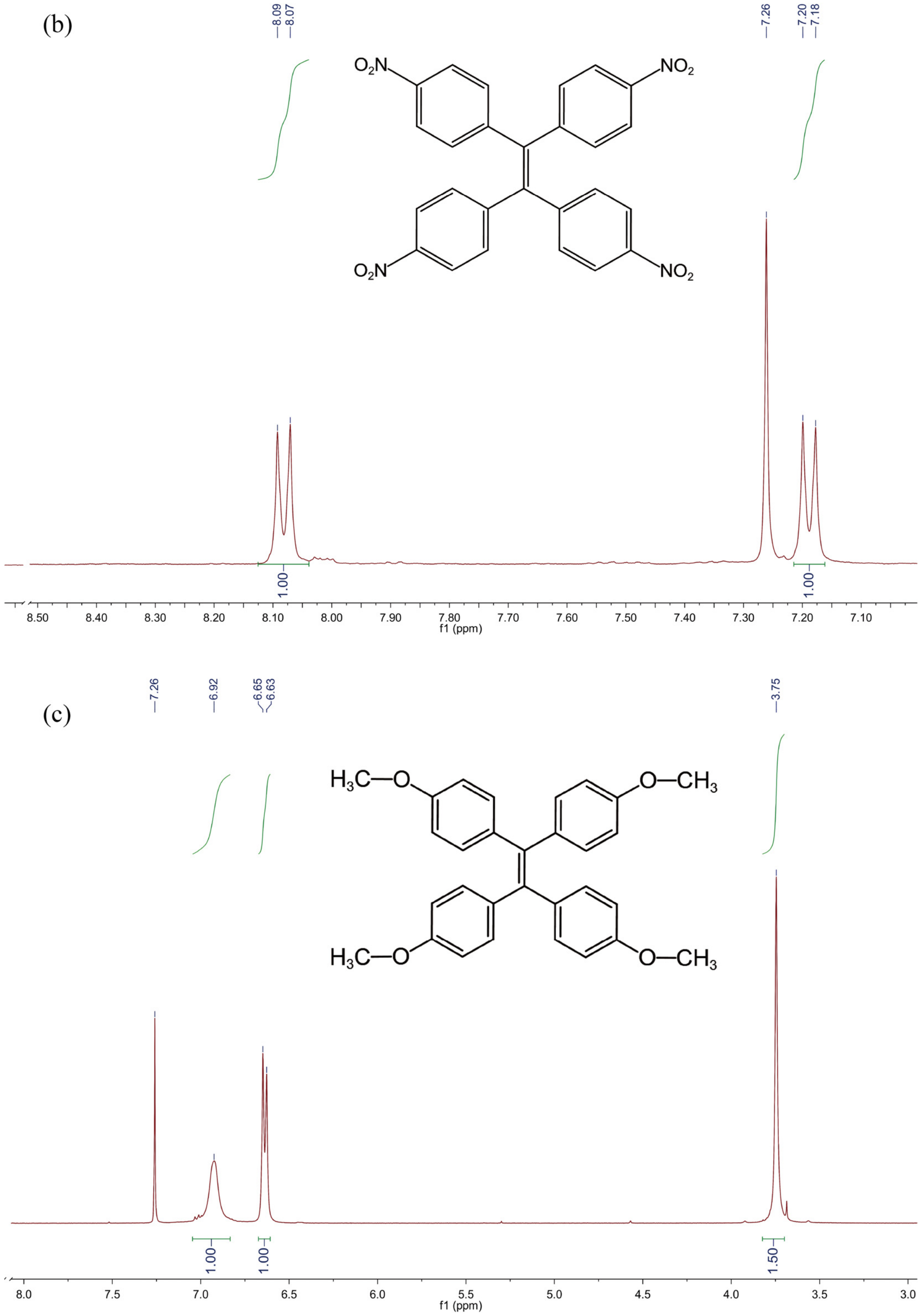

Figure S2. ^{1}H NMR spectrum of (**a**) TPE, (**b**) TPE-4NO2, (**c**) TPE-4OCH$_3$ in CDCl$_3$.

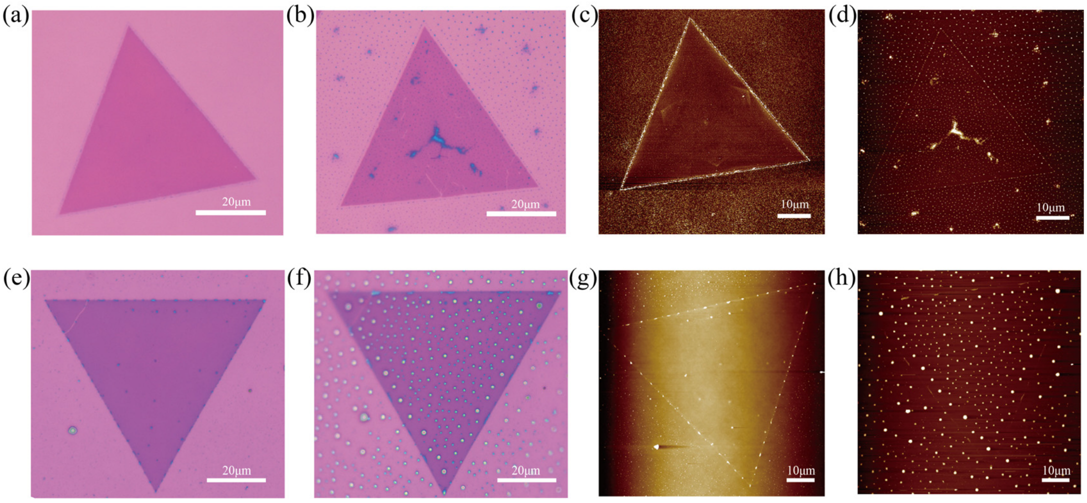

Figure S3. (**a–d**). Images of OM and AFM before and after TPE-4NO_2 doping. (**e–h**). Images of OM and AFM before and after TPE-4OCH_3 doping.

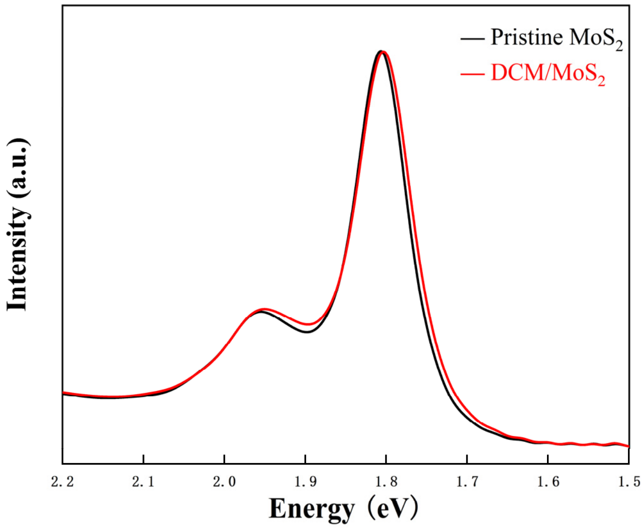

Figure S4. PL spectra of monolayer MoS_2 before and after soak in DCM for 12 hours.

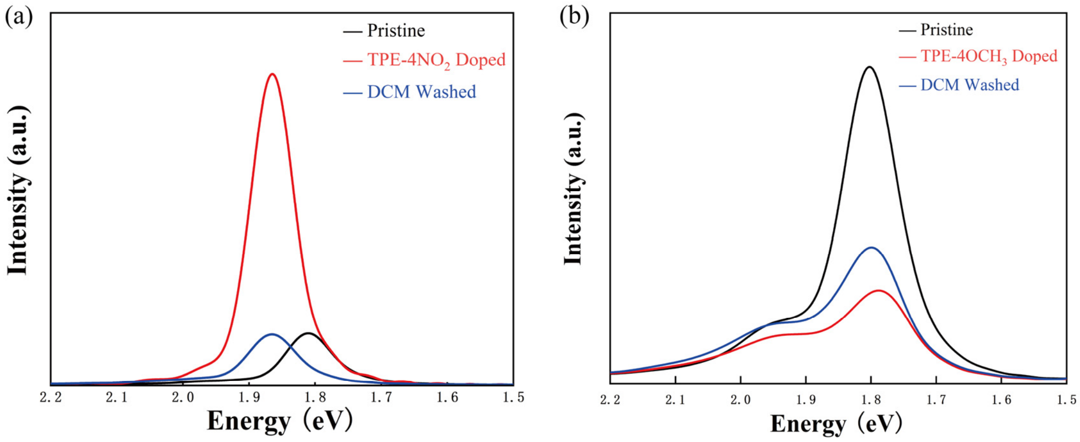

Figure S5. PL spectra of TPE-4NO_2 (**a**) and TPE-4OCH_3 (**b**) doped of immersion in dichloromethane (DCM) 12 hours.

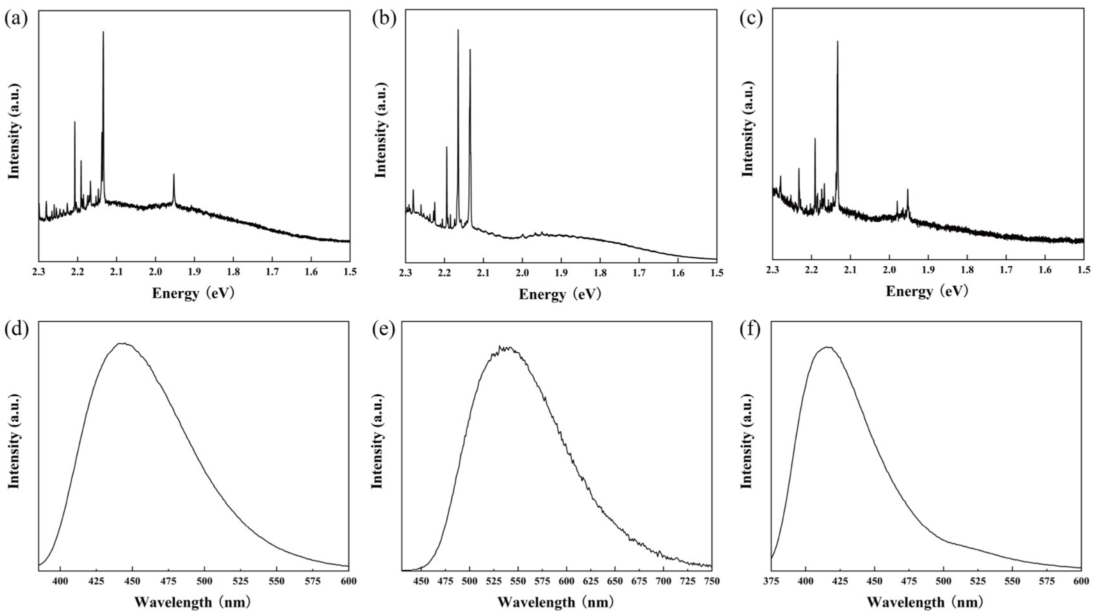

Figure S6. (**a**–**c**) PL spectra of TPE, TPE-4NO2 and TPE-4OCH3 molecular. (**d**–**f**) Fluorescence spectra TPE, TPE-4NO2 and TPE-4OCH3 molecular, excitation wavelength is at 365nm.

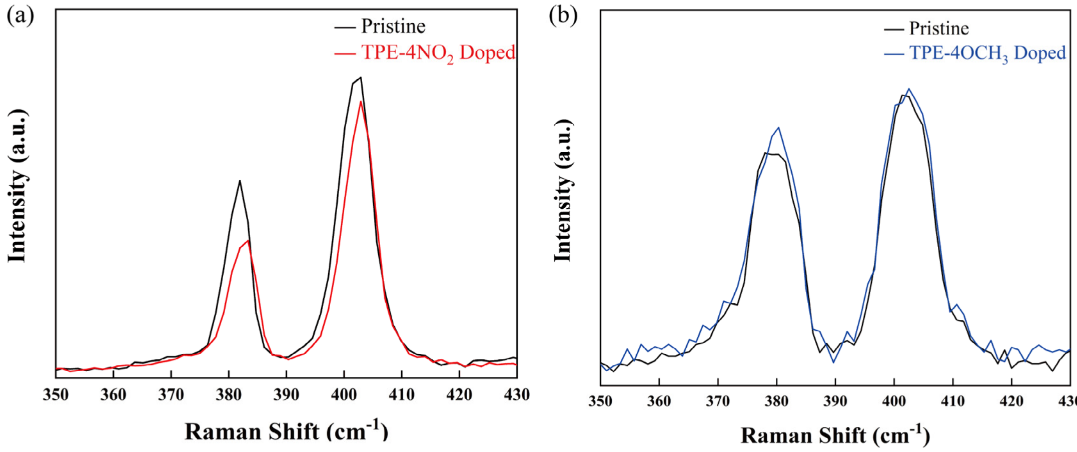

Figure S7. Raman spectra of TPE-4NO2 (**a**) and TPE-4OCH3 (**b**) doped.

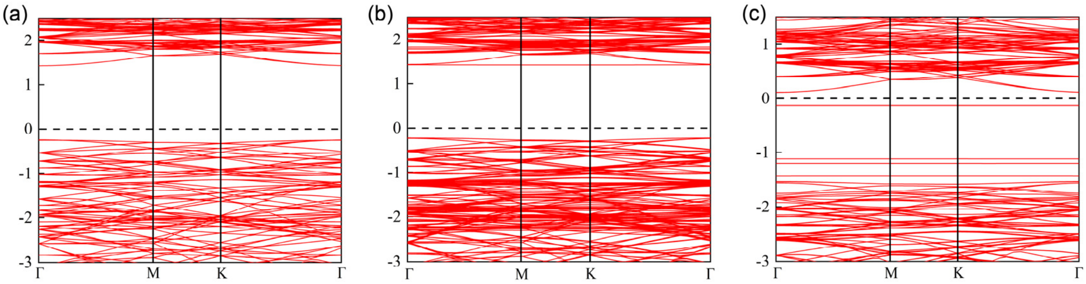

Figure S8. The electronic band structure of (**a**) the monolayer MoS_2, (**b**) MoS_2/TPE-4NO2, (**c**) MoS_2/TPE-4OCH3.
